# Salmonella enteritidis PT6: another egg-associated salmonellosis?

**DOI:** 10.3201/eid0404.980421

**Published:** 1998

**Authors:** M R Evans, W Lane, C D Ribeiro

**Affiliations:** Bro Taf Health Authority, Cardiff, United Kingdom. meirion.evans@bro-taf-ha.wales.nhs.uk

## Abstract

Salmonella Enteritidis phage type 6 (PT6) increased dramatically in the United Kingdom during 1997. The sharp rise suggests that PT6 contamination has spread rapidly throughout a basic food commodity; however, the source and food vehicle remain unknown. We present evidence from three outbreaks suggesting a possible link between PT6 and eggs. Poor documentation of the egg supply network continues to pose problems for public health investigators. Thorough investigation of all future PT6 outbreaks and case-control studies of sporadic infections are needed to confirm the etiology of PT6 infection.


### *Salmonella* Enteritidis PT6: Another Egg-Associated Salmonellosis?

**Meirion R. Evans,* Will Lane,† and C. Donald Ribeiro‡**
*Bro Taf Health Authority, Cardiff, United Kingdom;
†Cardiff Environmental Protection, Cardiff, United Kingdom; and
‡Cardiff Public Health Laboratory, Cardiff, United Kingdom

*Salmonella* Enteritidis phage type 6 (PT6) increased dramatically in the United Kingdom during 1997. The sharp rise suggests that PT6 contamination has spread rapidly throughout a basic food commodity; however, the source and food vehicle remain unknown. We present evidence from three outbreaks suggesting a possibile link between PT6 and eggs. Poor documentation of the egg supply network continues to pose problems for public health investigators. Thorough investigation of all future PT6 outbreaks and case-control studies of sporadic infections are needed to confirm the etiology of PT6 infection.

Nearly 10 years ago, evidence from both sides of the Atlantic first implicated eggs in the sharp increase during the 1980s of food poisoning due to *Salmonella* Enteritidis (1,2). In the United Kingdom and Western Europe, the predominant phage type (PT) responsible for eggborne *Salmonella* infection is PT4 (3). In the United States, although PT8 and PT13a are the most common phage types, no single phage type is more likely to be associated with eggborne infection (4). We report the possible emergence of a new egg-associated *Salmonella* phage type in the United Kingdom.

During 1997, we investigated three *Salmonella* Enteritidis PT6 food poisoning outbreaks in Cardiff, Wales. In outbreak 1, five of approximately 200 staff of an education establishment who regularly used a staff canteen reported diarrhea; three were positive for *S.* Enteritidis PT6. All five had eaten fish cakes from a batch of 18 served at the canteen; no illness was reported by other staff, nearly all of whom had eaten the alternative meal. Fish cakes, made with fish and reconstituted dried mashed potatoes, were shaped by hand, coated with raw shell egg, dipped in breadcrumbs, and shallow-fried in oil. When we reproduced the cooking process, as described in a previous outbreak (5), we found that fish cakes were too thick to be properly cooked by the shallow-frying method. The temperature varied greatly both between fish cakes and between parts of the same fish cake. Although the top and bottom of the fish cake were cooked, the egg coating on the sides had insufficient contact with the frying oil to achieve adequate cooking temperature.

Outbreak 2 occurred in a nursing home accommodating 99 residents on four floors. Two residents from different floors and two staff (a cook and a health-care worker) were initially confirmed with *S.* Enteritidis PT6 infection. The only common factor linking the residents was a pureed food diet. When we screened all 10 residents on pureed food diets and a sample of 30 residents on normal diets, we found four other *S.* Enteritidis PT6 cases, all in patients on pureed food diets. The health-care worker was probably infected by secondary spread. The cook's illness began 4 days after the index patient's, which suggested that the cook was infected from a common food source and had not in fact been the cause of the outbreak. Raw shell egg was regularly used in the kitchen; pureed food may have been cross-contaminated from a mixing bowl used for both raw and pureed food.

In outbreak 3, eight customers of the same small Italian restaurant had *S.* Enteritidis PT6

Address for correspondence: Meirion R. Evans, Public Health Directorate, Bro Taf Health Authority, Cathays Park, Cardiff, CF1 3NW, United Kingdom; fax: 44-1222-371104; e-mail:meirion.evans@bro-taf-ha.wales.nhs.uk.

food poisoning. All had eaten the dessert tiramisu during a 5-day period. Two batches of tiramisu containing raw shell egg were implicated, but neither was available for microbiologic examination. However, a layered cassata ice cream also containing raw egg was tested, and the top layer, made the same day as the first batch of tiramisu, was *S.* Enteritidis PT6–positive. Kitchen inspection indicated that the second batch of tiramisu was probably cross-contaminated when the same whisks and mixing bowls used to make the first batch were reused.

PT6 is a hitherto uncommon phage type in the United Kingdom. In Cardiff, one case not associated with foreign travel was reported in 1994 and in 1995, and two in 1996. During 1997, in addition to the outbreaks described, 33 sporadic cases were reported in Cardiff. In Wales as a whole, reports of *S.* Enteritidis PT6 rose from 23 in 1995 and 43 in 1996 to 279 in 1997, while reports of PT4 remained constant (PHLS Communicable Disease Surveillance Centre [Wales], unpub. data) (Figure). More than 1,000 cases of *S.* Enteritidis PT6 were reported in England and Wales in 1996, almost twice the number in 1995, making it the second most common phage type after PT4 (6). Most cases appear sporadic and acquired within the United Kingdom. Poultry meat and shell eggs were implicated in three of the six outbreaks reported in 1996 (2). The largest outbreak involved 49 persons, of whom 13 had laboratory confirmed

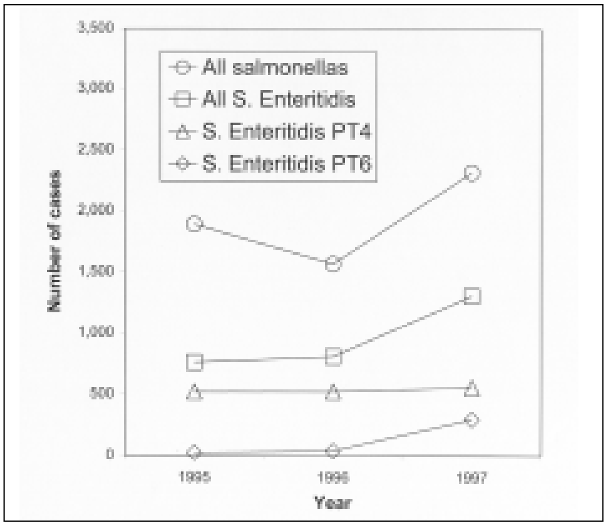

Figure. Salmonella isolates reported in Wales by serotype and phage type, 1995-1997. Source: PHLS Communicable Disease Surveillance Centre (Wales).

*S.* Enteritidis PT6 (7). Eating egg sandwiches served at a buffet meal was strongly statistically associated with illness, and *S.* Enteritidis PT6 was isolated from several environmental samples from the kitchen where the food had been prepared. However, the investigation did not show whether or not shell eggs used in the sandwiches were the original source or had been cross-contaminated.

The three *S.* Enteritidis PT6 outbreaks described occurred in very different settings: a work canteen, a nursing home, and a public restaurant. Although the evidence is circumstantial, our reports suggest a possible link between *S.* Enteritidis PT6 and eggs. Traceback of eggs was particularly difficult, both because of poor egg labeling and because of the complex network of suppliers and distributors involved. As far as could be ascertained, the eggs associated with the three outbreaks were from different sources, which suggests a general egg contamination problem; if this is the case, the egg contamination signals a complete failure of measures to eradicate salmonellae from poultry-breeding flocks. Alternatively, egg contamination may be restricted to one particular sector of the egg industry. Salmonella-contaminated eggs imported into the United Kingdom have been associated with particular packing stations (8). Traceback investigations of eggs in the United Kingdom continue to pose difficulties for public health investigators because of deficiencies in documentation kept on the egg distribution network. Identifying the source of egg-related outbreaks requires better egg labeling and documentation at all levels of the supply chain—from farmer through distributor to the point of retail sale.

The increase in *S.* Enteritidis PT6 could also be explained by molecular change in the previously dominant PT4. However, PTs 4 and 6 are well established distinct types whose type strains have remained unchanged for many years (5); PT6 is probably not replacing PT4 since no concomitant decrease in PT4 incidence has been observed. The sharp rise in PT6 incidence in the United Kingdom over the past 18 months therefore suggests that this phage type has found a new ecologic niche and that contamination has spread rapidly throughout a basic food commodity. The source and food vehicle remain unknown. All *S.* Enteritidis PT6 outbreaks should be throughly investigated, and case-control studies are needed to establish the etiology of sporadic PT6 infections.

Dr. Evans is a consultant in communicable disease control, Public Health Directorate, Bro Taf Health Authority, Cardiff, and honorary consultant epidemiologist, Public Health Laboratory Service Communicable Disease Surveillance Centre (Wales). His research interests include the epidemiology of foodborne disease, childhood immunization, and travel health.

### References

1. Coyle EF, Palmer SR, Ribeiro CD, Jones HI, Howard AJ, Ward L, et al. *Salmonella enteritidis* phage type 4 infection: association with hen's eggs. Lancet 1988;2:1295-7.
2. St. Louis ME, Morse DL, Potter ME, De Melfi TM, Guzewich JJ, Tauxe RV, et al. The emergence of grade A eggs as a major source of *Salmonella enteritidis* infections. JAMA 1988;259:2103-7.
3. Rampling A. *Salmonella enteritidis* five years on. Lancet 1993;342:317-8.
4. Mishu B, Koehler J, Lee LA, Rodrigue D, Brenner FH, Blake P, et al. Outbreaks of *Salmonella enteritidis* infections in the United States, 1985 – 1991. J Infect Dis 1994;169:547-52.
5. Evans MR, Hutchings PG, Ribeiro CD, Westmoreland D. A hospital outbreak of salmonella food poisoning due to inadequate deep-fat frying. Epidemiol Infect 1996;116:161-7.
6. PHLS Communicable Disease Surveillance Centre. Salmonella infections, England and Wales: reports to the PHLS (salmonella data set). Commun Dis Rep CDR Wkly 1997;7:208.
7. Holtby I, Tebbitt GM, Grunert E, Lyle HJ, Stenson MP. Outbreak of *Salmonella enteritidis* phage type 6 infection associated with food items provided at a buffet meal. Commun Dis Rep CDR Rev 1997;7:87-90.
8. de Louvois J. Salmonella contamination of eggs. Lancet 1993;342:366-7.
9. Stanley J, Jones CS, Threlfall EJ. Evolutionary lines among *Salmonella enteritidis* phage types are identified by insertion sequence IS*200* distribution. FEMS Microbiol Lett 1991;82:83-90.
